# The Identification by Exome Sequencing of Candidate Genes in *BRCA*-Negative Tunisian Patients at a High Risk of Hereditary Breast/Ovarian Cancer

**DOI:** 10.3390/genes13081296

**Published:** 2022-07-22

**Authors:** Dorra BenAyed-Guerfali, Chamseddine Kifagi, Wala BenKridis-Rejeb, Nihel Ammous-Boukhris, Wajdi Ayedi, Afef Khanfir, Jamel Daoud, Raja Mokdad-Gargouri

**Affiliations:** 1 Center of Biotechnology of Sfax, University of Sfax, Sidi Mansour Street Km 6, BP 1177, Sfax 3038, Tunisia; dorra.benayed@cbs.rnrt.tn (D.B.-G.); chamseddine.kifagi@gmail.com (C.K.); ammousnihel@yahoo.fr (N.A.-B.); wajdi.ayadi.cbs@gmail.com (W.A.); 2 Department of Medical Oncology, Habib Bourguiba Hospital, Sfax 3002, Tunisia; walabenkridis@yahoo.fr (W.B.-R.); afefkhanfir@yahoo.fr (A.K.); 3 Department of Radiotherapy, Habib Bourguiba Hospital, Sfax 3002, Tunisia; jameldaoud50@gmail.com

**Keywords:** *BRCA1/BRCA2* genes, hereditary breast/ovarian cancer, exome sequencing, germline variants, candidate genes, pathogenic variants

## Abstract

(1) Background: Germline variants in *BRCA1/BRCA2* genes explain about 20% of hereditary breast/ovarian cancer (HBOC) cases. In the present paper, we aim to identify genetic determinants in *BRCA*-negative families from the South of Tunisia. (2) Methods: Exome Sequencing (ES) was performed on the lymphocyte DNA of patients negative for *BRCA* mutations from each Tunisian family with a high risk of HBOC. (3) Results: We focus on the canonical genes associated with HBOC and identified missense variants in DNA damage response genes, such as *ATM*, *RAD52*, and *RAD54*; however, no variants in *PALB2*, *Chek2*, and *TP53* genes were found. To identify novel candidate genes, we selected variants harboring a loss of function and identified 17 stop-gain and 11 frameshift variants in genes not commonly known to be predisposed to HBOC. Then, we focus on rare and high-impact genes shared by at least 3 unrelated patients from each family and selected 16 gene variants. Through combined data analysis from MCODE with gene ontology and KEGG pathways, a short list of eight candidate genes (*ATM*, *EP300*, *LAMA1*, *LAMC2*, *TNNI3*, *MYLK*, *COL11A2*, and *LAMB3*) was created. The impact of the 24 selected genes on survival was analyzed using the TCGA data resulting in a selection of five candidate genes (*EP300*, *KMT2C*, *RHPN2*, *HSPG2*, and *CCR3)* that showed a significant association with survival. (4) Conclusions: We identify novel candidate genes predisposed to HBOC that need to be validated in larger cohorts and investigated by analyzing the co-segregation of selected variants in affected families and the locus-specific loss of heterozygosity to highlight their relevance for HBOC risk.

## 1. Introduction

Breast cancer (BC) is the most prevalent cancer worldwide and the second leading cause of death by cancer in women [1,2]. In Tunisia, the incidence of BC is about 3092 new cases/year affecting more often young women (<35 years old), and with more aggressive clinical behavior [3,4]. Ovarian cancer (OC) is less frequent with an incidence of 284 new cases/year [5]. 

Hereditary breast and ovarian cancer (HBOC) is an inherited disorder in which the risk of breast and ovarian cancers is higher compared to the general population. About 5 to 10% of breast cancers and 10 to 15% of ovarian cancers can be attributed to the hereditary form [6]. Two major predisposition genes, *BRCA1* and *BRCA2*, account for approximately 25% of HBOC cases worldwide [7,8]. Heterozygote individuals for pathogenic variants in *BRCA1* have a 72% and 44% increased risk of BC and OC, respectively, by the age of 80 years. Concerning *BRCA2*, the risk of BC and OC at the age of 80 years was 69% and 17%, respectively [9]. Individuals harboring pathogenic variants in these genes also have an increased risk for developing other malignancies, including melanoma, prostate, and pancreatic cancer, suggesting an important role for these genes in cancer predisposition [10].

It was well documented that the frequency of *BRCA* mutations varies between populations [11]. In a recent meta-analysis that included 14 Arab countries, the authors reported that the prevalences of *BRCA1* and *BRCA2* germline mutations were 11% and 17%, respectively [12]. In Tunisia, the prevalence of *BRCA1/BRCA2* deleterious mutations varies from 25% to 18% in HBOC patients [13,14,15,16]. In our recent study, using NGS, we found that among 113 patients, 18 (15.9%) harbored germline *BRCA* mutations [17]. Furthermore, recurrent mutations have been reported in the North African population, such as *BRCA2*-c.1310_1313delAAGA [17,18,19,20]. However, some mutations are likely to be specific to the Tunisian population and more precisely to patients from the North-East, such as *BRCA1*-c.211dupA [13,14,15,21], or from the South, such as the *BRCA1*-c.5030_5033delCTAA and the *BRCA2*-c.17_20 delAAGA mutations [17].

Despite the extensive research, the genetic etiology of a high percentage of HBOC cases (more than 50%) remains unknown [22,23]. Thanks to next-generation sequencing (NGS), in particular, whole-exome sequencing (WES) or targeted-exome sequencing (ES) approaches, researchers have managed to identify novel variants predisposed to HBOC [24,25]. However, only few WES or ES studies have been conducted on Tunisian families. The study conducted by Hamdi et al. investigated, by WES, two affected sisters from a *BRCA*-negative Tunisian family with a high risk of HBOC, and showed that *MMS19*, *DNHA3*, *POLK*, and *KATB6* were interesting breast cancer candidate genes [26]. Furthermore, a novel truncating mutation in the *RCC1* gene was found in Tunisian breast cancer patients using WES associated with genotyping, suggesting that *RCC1* is a novel breast cancer susceptibility gene [16].

In the present study, we apply ES to Tunisian HBOC families with an unknown genetic etiology to identify novel candidate genes predisposed to HBOC. The findings are also explored for cancer patients in The Cancer Genome Atlas (TCGA).

## 2. Materials and Methods

### 2.1. Materials

We selected 113 families with a high risk of HBOC between 2016–2019 in the Medical Oncology Unit of the CHU Habib Bourguiba of Sfax, Tunisia. The criteria used to include patients in the present study were: (1) the presence of at least three related first- or second-degree breast cancer cases; (2) breast cancer in young patients aged less than 35 years, (3) the presence of male breast cancer among first- or second-degree patients, (4) the presence of at least two cases of breast or ovarian cancer, regardless of age, and at least one case of prostate cancer in a related first- or second-degree patient. Among the 113 patients, 95 were negative for germline *BRCA* mutations, and we selected 8 unrelated patients with a high risk of HBOC for exome sequencing (ES). 

### 2.2. DNA Extraction and Exome Sequencing 

Genomic DNA was isolated from 0.2 mL of peripheral blood obtained from patients from each selected family using the “QIAamp DNA Blood Mini Kit” (Qiagen), following the manufacturer’s instructions. Isolated DNA was quantified by Qubit 3.0 fluorometric quantitation (Thermo Fisher Scientific, USA). An aliquot of 50 ng of DNA was used to prepare the library according to the manufacturer’s instructions (Illumina, USA). Exome capture was performed using the Trusight One Sequencing Panel Kit that provides comprehensive coverage of 4800 disease-associated genes (Illumina, USA). The samples were subjected to paired-end sequencing using the Illumina Miseq platform with a 151-bp read length using a MiSeq Reagent Kit v3 (Illumina, USA).

Exome DNA sequences were mapped to their location in the building of the human genome (hg19/b37) using the Burrows–Wheeler Aligner (BWA) package. The total PF read was 17,979,434 and the Q30 was 90.3%. The mean region coverage depth was 110.9 and the 30X depth of sequencing coverage was 89.5%. Data were analyzed using the BaseSpace Variant Interpreter (https://basespace.illumina.com (accessed on 6 November 2019).

### 2.3. Functional Annotation and Variants’ Prioritization

To identify pathogenic variants (PVs), we applied the following criteria: (i) variants found in a heterozygous state; (ii) variants located in coding region, 5′-3′UTR, splicing; (iii) rare variants with a minor allele frequency (MAF) ≤ 0.01% in both 1000 genomes and gnomAD; and (iv) missense variants predicted as damaging by SIFT (scores < 0.05), PolyPhen-2 (score > 0.9) and CADD tool (score ≥ 25).

Global Network is built from all the mutated genes reported in our study and protein–protein interaction (PPI) data using Cytoscape software and related plugin “STRING” [27,28]. To detect densely connected regions and clusters in the “Global Network”, a plug-in of Cytoscape v3.9.0 called Molecular Complex Detection (MCODE) was used. Those modules/clusters are often expected to be protein complexes and parts of pathways. The criteria settings were set as follows: node score cutoff = 0.1; K-core = 2; and fluff and degree of cutoff = 2. Gene ontology (GO) and the Kyoto Encyclopedia of Genes and Genomes (KEGG) pathway analyses were realized through another Cytoscape plugin: “ClueGo” [29].

### 2.4. Survival Analysis

The survival analysis of 8988 breast cancer samples from 13 studies was performed using the cBioPortal (https://www.cbioportal.org/ platform (accessed on 26 August 2021) [30,31]. The log-rank test of the SPSS 20 version was used to determine the significance of the differences in the overall survival (OS) and disease-free survival (DFS) probabilities between the 2 groups; *p* < 0.05 was considered as statically significant.

## 3. Results

### 3.1. Clinical Characteristics of BRCAness Patients 

**Figure 1:**
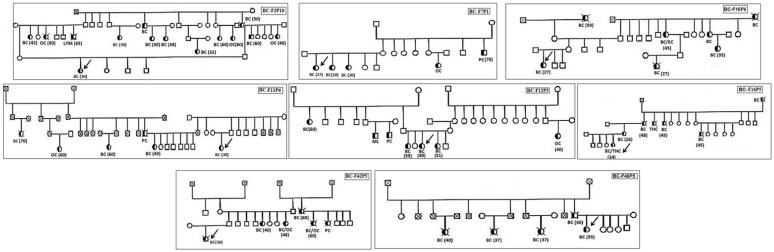
Pedigrees of the families selected for exome sequencing. The arrow indicates the sequenced patients from each family. Available age at diagnosis of breast cancer cases is indicated.

In our previous work, we screened by NGS all exons of the *BRCA1* and *BRCA2* genes in 113 Tunisian patients with HBOC. Deleterious *BRCA* mutations were identified in 18 patients (15.9 %) [17]. Among the 95 BRCA-negative cases, we selected eight patients from unrelated families with a high risk of HBOC. The selected families had several affected members with various types of cancers, such as breast, ovarian, prostate, thyroid, and melanoma. We also mentioned that two families (BCF2 and BCF46) had cases of male breast cancer (Figure 1, Table 1). The ages of the selected patients were between 24 and 55 years and two cases presented triple-negative breast cancer (TNBC). The clinical characteristics of the patients are summarized in Table 1.

### 3.2. Exome Sequencing and Data Analysis 

**Figure 2:**
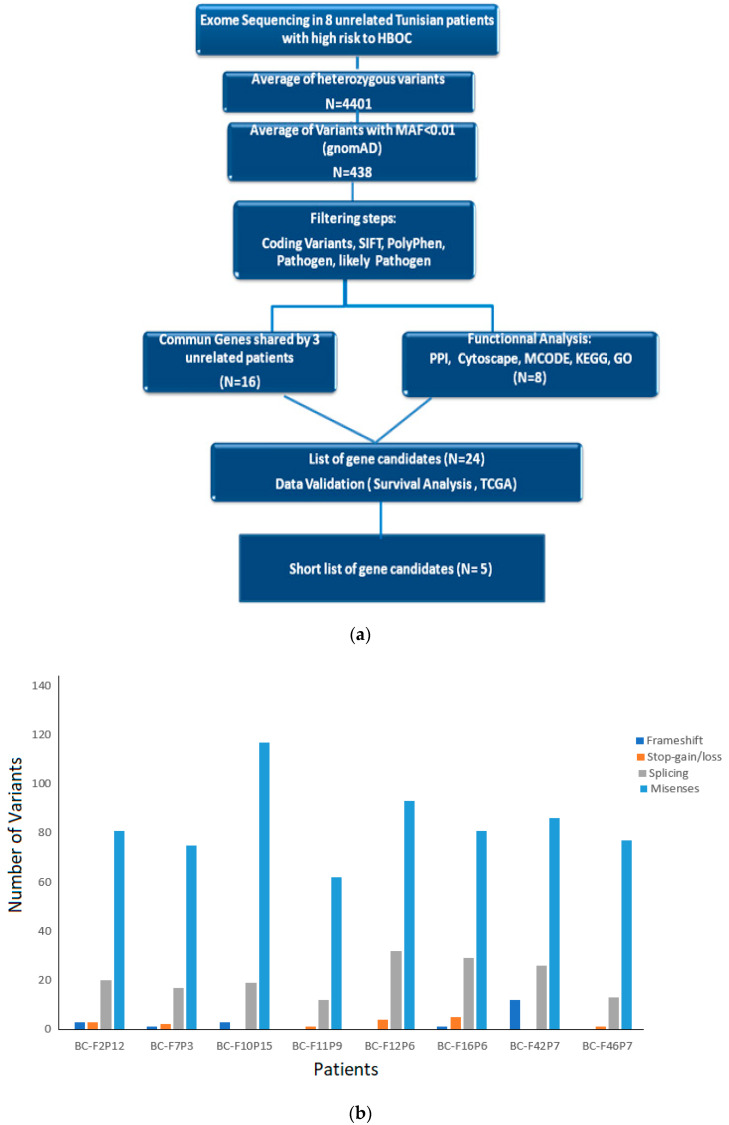
(**a**) Workflow showing the different steps performed for ES data analysis. (**b**) Histogram showing the number of variants identified by exome sequencing in each patient from the selected families.

Exome sequencing (ES) was performed for eight BRCAness patients to identify candidate genes associated with the malignancy. Before applying filters, the total number of variants varied between 5607 and 7802 and the heterozygous variants varied between 3413 and 5248. A workflow showing the different steps performed during the analysis of the ES data is presented in Figure 2a. Missenses mutations were the most frequently found compared to frameshift, stop-gain/loss, or splicing (Figure 2b). Splicing variants included donor site, acceptor site, as well as splicing region in intron. For example, the BC-F12 family had the highest number of splicing variants as we identified one splice acceptor variant (c.197-2A>G) in the AIF gene and one splice variant donor (c.4636+1G>T) in the SLX4 gene; both are classified as “likely pathogen” in ClinVar. The other splicing variants are located in the intronic splicing region.

In the first step of our analysis, we focused on the canonical genes predisposed to HBOC, such as *ATM*, *PALB2*, *Chek2*, *CDH1*, *PTEN*, and *TP53*, but no variants in these genes were detected except for two missenses in the *ATM* gene, c.1810C>T; p.Pro604Ser and c.6115G>A; p.Glu2039Lys, which were identified in two patients from the BC-F15 and BC-F16 families diagnosed with stage-II BC at the ages of 30 and 27 years, respectively. 

Evidently, the c.6115G>A variant has been reported as likely pathogenic in the ClinVar database and as VUS according to the ACMG classification, while the c.1810C>T variant has been reported as presenting conflicting interpretations of pathogenicity in ClinVar. Then, we focused on genes traditionally associated with breast/ovarian cancer or other malignancies, and identified 23 missenses, 2 splicing, and 2 UTR variants mainly affecting genes involved in DNA repair, such as *RAD51*, *RAD54B*, *PMS1*, *FANCD2*, *XRCC1*, and *BLM* (Table 2). The same *RAD52* variant (c.661C<G, p.Gln221Glu) was identified in two unrelated patients (BC-F10P6 and BC-F16P5; Table 2). About 50% of the identified variants were classified as VUS according to ClinVar and Varsome (Table 2).

### 3.3. Identification of Potential Gene Candidates Predisposed to HBOC

In the next step of our analysis, we selected genes harboring loss-of-function (LoF) variants (stop-gain, splice site, and frameshift), because these variant types have an impact on protein function and are commonly linked to disease susceptibility (Richards et al., 2015). In this step, we identified 17 stop-gain and 11 frameshift variants affecting genes not commonly known as to be predisposed to HBOC (Table 3). Interestingly, among the high-risk variants listed, two frameshift variants were recurrent in unrelated patients, namely, c.1200delA; p.Lys400AsnfsTer15 in *BHMT* found in BC-F7P1 and BC-F10P6, and c.2626-2629delTTTG; p.Phe876LysfsTer16 in *SH3PXD2B* shared by BC-F2P10 and BC-F42P5 patients (Table 3). In addition, several stop-gain variants were identified in different genes, such as *APOBEC3B*, *CERKL*, *SUMG1*, and *PIKFYVE* (Table 3). 

**Figure 3:**
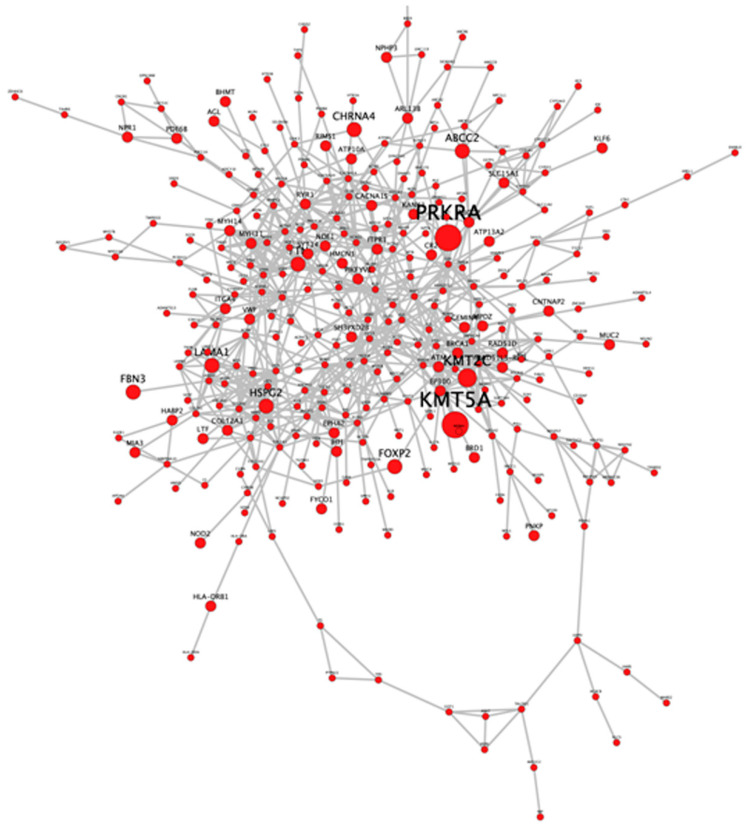
Interactome showing the protein–protein interactions deduced from the exome sequencing data. The size of the red dots is proportional to the number of times that the gene was identifiedin patients.

**Figure 4:**
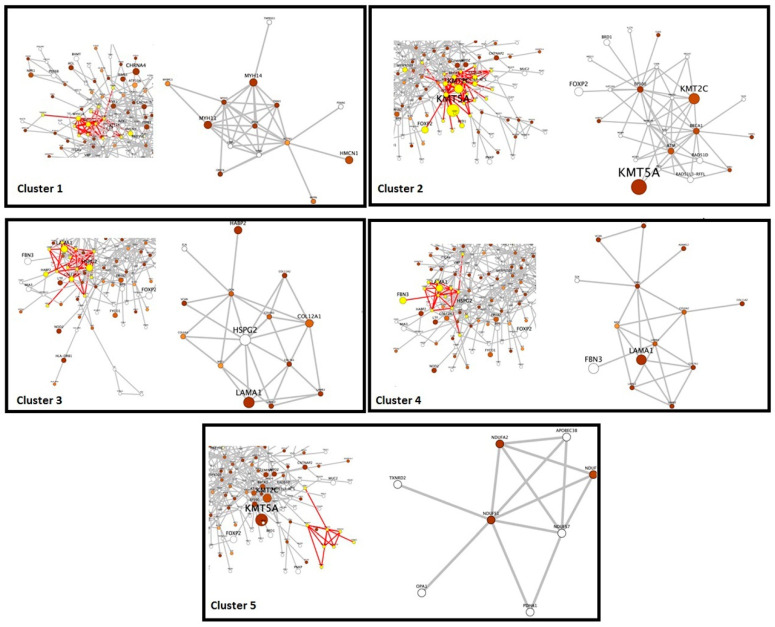
MCODE analysis identified 5 clusters: Cluster 1: 20 genes (LAMA1, LAMC2, LAMA5…), Cluster 2: 12 genes (MYH11, MYH13, MYH14…), Cluster 3: 33 genes (EP300, ATM, XRCC1…), Cluster 4: 12 genes (ABCG2, ABCC2, CFTR…), Cluster 5: 12 genes (PDE11A, CNGB1, PRKACA…). For more details, see supplementary data Table S1.

**Figure 5:**
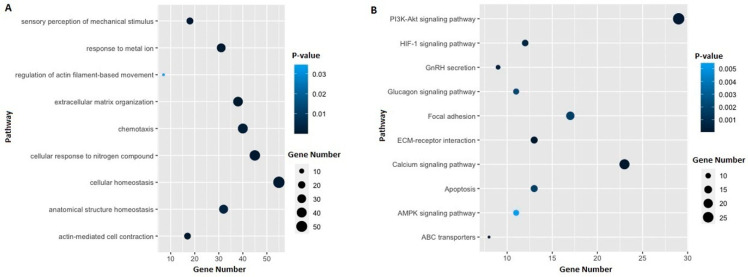
GO and KEGG bubble of mutant genes of analyzed samples. (**A**) Bubble plot of the Gene Ontology (GO) analysis showing the top 9 significant pathways. Bubble color and size correspond to the P-value and gene number enriched in the pathway. (**B**) Bubble plot of the KEGG (Kyoto Encyclopedia of Genes and Genomes) analysis showing the top 10 significant pathways. Bubble color and size correspond to the P-value and gene number enriched in the pathway.

Furthermore, we performed the global network analysis to identify the protein–protein interaction (PPI) between proteins deduced from the ES data. Out of a total of 474 candidate genes, 336 PPIs were identified (Figure 3). Through a functional analysis based on MCODE, we selected the five most significant clusters that were involved in protein complexes and signaling pathways (Figure 4). The list of genes in each cluster is available in the Supplementary Materials, Table 1. Furthermore, gene ontology (GO) and the Kyoto Encyclopedia of Genes and Genomes (KEGG) pathway analysis of the selected genes were performed, and we found that they were mainly involved in extracellular matrix organization, actin-mediated cell contraction, response to metal ions, cellular response to nitrogen compounds, and cellular homeostasis (Figure 5a). Additionally, the results of the KEGG pathway analysis show that gene candidates are distributed in the ECM-receptor interaction, calcium signaling pathway, PI3K-Akt signaling pathway, ABC transporters, GnRH secretion, HIF-1 signaling pathway, apoptosis, focal adhesion, glucagon signaling pathway, and AMPK signaling pathway (Figure 5b). The combined analyses from MCODE with gene ontology and KEGG pathways led us to retain a short list of eight candidate genes (*ATM*, *EP300*, *LAMA1*, *LAMC2*, *TNNI3*, *MYLK*, *COL11A2*, and *LAMB3*). The selected genes were involved in at least 2 among the selected top 10 pathways, and the corresponding variants displayed significant Sift and PolyPhen scores.

**Figure 6:**
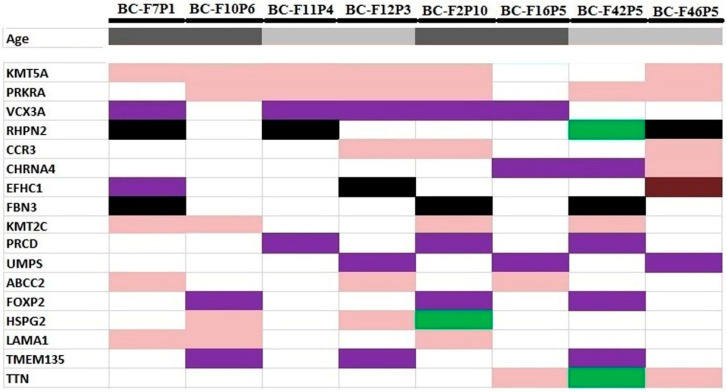
Variants identified in the candidate genes selected trough functional analysis and that are shared by at least 3 unrelated patients. In light pink: missense variants; in black: splice acceptor/donor variants; in purple: 3′UTR variants; in brown: 5′UTR variants; and in green: more than one variant. Information about age at diagnosis: in dark gray: diagnosis less than or equal to 40 years of age; in light grey: more than or equal to 40 years of age.

On the other hand, we selected gene variants that were shared by at least 3 patients from unrelated families and listed the 16 common genes: *KMT5A*, *PRKRA*, *VCX3A*, *RHPN2*, *CCR3*, *CHRNA4*, *EFHC1*, *FBN3*, *KMT2C*, *PRCD*, *UMPS ABCC2*, *FOXP2*, *HSPG2*, *TMEM135*, and *TTN* (Figure 6). It is interesting to note that we found the same variant in the *KMT5A* (c.995T>C), *RHPN2* (c.1225+5G>A), *CCR3* (c.727A>G), and *KMT2C* (c.2645T>C) genes in at least three unrelated patients. Based on the TCGA data, the most frequently altered gene in breast cancer was *KMT2C* (12%), and the *TTN* gene (17%) in ovarian cancer. A table listing the variants identified in the selected gene candidates is available in the Supplementary Materials (Supplementary Table S2).

### 3.4. Survival Analysis

**Figure 7:**
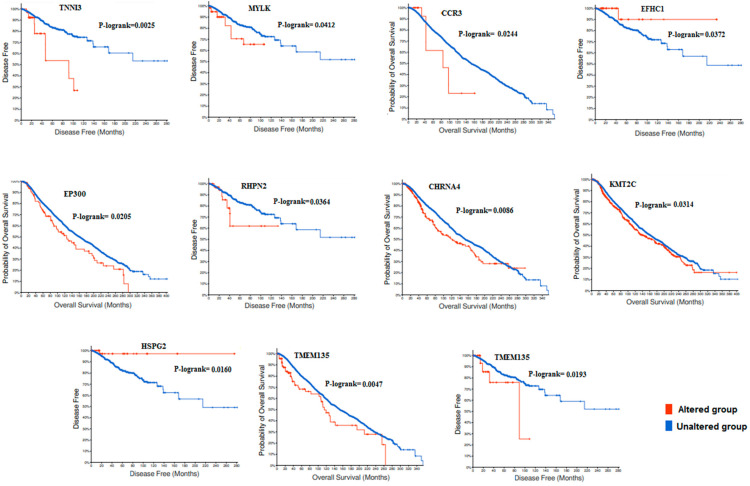
Kaplan–Meier plots showing significant associations between the selected candidate genes and the overall survival or disease-free survival using the TCGA data.

To investigate the impact of the retained genes on the survival rates, we used the TCGA data including 14 studies conducted between 2012 to 2020 to associate the 25 candidate genes with overall survival (OS) and disease-free survival (DFS). Kaplan–Meier curves showed that only 11 genes among 25 were significantly associated with OS or DFS (Figure 7). With concerns of genes related to BOC as reported by the literature data, significant associations were observed between the survival rate and *EP300* (P logrank: 0.0205), *KMT2C* (P logrank: 0.0314), *RHPN2* (P logrank: 0.0364), *HSPG2* (P logrank: 0.016), and *CCR3* (P logrank: 0.0244).

## 4. Discussion

Next-generation sequencing (NGS) technology has revolutionized the clinical approach for genetic testing in oncology. Whole-exome sequencing (WES) is increasingly used for screening HBOC patients for clinical applications and precision therapy [25,32,33]. Indeed, WES as well as multi-gene panels are powerful methods for the identification of pathogenic variants (PVs) in known HBOC-related genes and novel variants that might be associated with the disease [24,34,35,36,37,38]. Evidently, only few exome sequencing studies were conducted on BRCAness Tunisian families compared to other populations. As an example, Riahi et al. identified by WES a novel frameshift mutation in the *RCC1* gene encoding the regulator of chromosome condensation 1. This variant (c.1067_1086del19) has exclusively been found in Tunisian breast cancer patients [16]. Other candidate genes (*MMS19*, *DNHA3*, *POLK*, and *KATB6*) were identified in a BRCAness family from the North of Tunisia [26]. More recently, and using WES, Mighri et al. identified a rare exonic VUS on an *RAD50* gene (c.3647C>G, p.Ala1216Gly), a breast cancer susceptibility gene in a patient originating from the North-Eastern Tunisian region [21].

In our previous study, we reported that the contribution of the predisposing *BRCA1/BRCA2* genes was involved in 15.9 % of HBOC patients originating from the South of Tunisia [17]. To further investigate the genetic landscape of HBOC, we performed ES on eight *BRCA*-negative patients from unrelated families. Initially, we focused on the canonical genes predisposed to HBOC, such as *ATM*, *PALB2*, *Chek2*, *CDH1*, *PTEN*, and *TP53*, but no PV was found, except for two missense variants in the *ATM* gene that is involved in DNA double-strand-break repair mechanisms and considered as a moderate-penetrance gene [39,40]. Missense variants were identified in DNA repair genes, such as *RAD51*, *RAD54B*, *PMS1*, *XRCC1*, and *BLM*. Moreover, we noticed that the same variant in *RAD52* (Gln221Glu) was found in two patients from unrelated families. 

After investigating known breast cancer genes, we extended our analysis to select rare loss-of-function variants, and we identified 11 frameshift and 17 stop-gain variants affecting genes involved in various cellular functions but not traditionally associated with HBOC. Nevertheless, among these 28 genes, 10 have been previously reported as implicated in breast/ovarian carcinogenesis. Among these variants, the frameshift in *SH3PXD2B* (c.2626-2629delTTTG) and *BHMT* (c.1200delA) genes were found in two unrelated patients. SH3PXD2B (Tks4) is a scaffold protein that plays a critical role in the invasion and metastasis of various types of tumors as hepatocarcinoma, melanoma, and breast cancer [41]. In fact, it was well demonstrated that cancer cells develop invadopodia and podosomes composed of structural proteins, such as cortactin, Tks4, and Tks5, to facilitate their migration across the endothelial layer to invade distant tissues [42].

Betaine-homocysteine *S*-methyltransferase (*BHMT*) catalyzes the synthesis of methionine from betaine and homocysteine. A previous study showed that choline and betaine are tightly associated with breast carcinogenesis [43,44].

In addition to frameshift variants, stop-gain variants were also identified in several genes involved in various cellular functions, such as the APOBEC3B (Apolipoprotein B mRNA-editing enzyme, catalytic polypeptide-like) belonging to the family of APOBEC enzymes that are strong mutagenic factors in human cancer [45,46]. *APOBEC3B* has been described as a strong driver of breast cancer and associated with aggressive clinical and pathological features [47,48,49]. A stop-gain variant (c.1166C>T) in *APOBEC3B* was identified in a BC-F12P6 patient, diagnosed with BC at 47 years of age, and with 3 BC cases and 2 OC cases in this family.

On the other hand, we performed functional analysis based on MCODE and selected the top five significant clusters that are involved in protein complexes and signaling pathways. Furthermore, the gene ontology (GO) and Kyoto Encyclopedia of Genes and Genomes (KEGG) pathway analysis showed that the selected genes were mainly involved in extracellular matrix organization, actin-mediated cell contraction, the PI3K-Akt signaling pathway, ABC transporters, and HIF-1 signaling pathway. Finally, we selected a list of eight candidate genes (*ATM*, *EP300*, *LAMA1*, *LAMC2*, *TNNI3*, *MYLK*, *COL11A2*, and *LAMB3*) that were involved in at least two among the selected pathways and showing damaging effects predicted by Sift and PolyPhen tools. *LAMA1*, *LAMC2*, and *LAMB3* belong to the laminin family, extracellular glycoproteins that are essential components of membranes and involved in tissue differentiation, progression, and invasion [50,51].

In this study, we identified *LAMA1* missense variants in three unrelated patients and noted that the BC-F7P1 patients with metastatic BCs harbored missense mutations in *LAMA1*, *LAMC2*, and *LAMAC5* genes.

Furthermore, we focused on gene variants that were shared by at least three patients from unrelated families and listed the following genes: *KMT5A*, *PRKRA*, *VCX3A*, *RHPN2*, *CCR3*, *CHRNA4*, *EFHC1*, *FBN3*, *KMT2C*, *PRCD*, *UMPS ABCC2*, *FOXP2*, *HSPG2*, *TMEM135*, and *TTN*. We noticed that only a few of them have been previously described as related to breast/ovarian cancer, such as *KMT2C*, *FOXP2*, *RHPN2*, and *HSPG2*.

Regarding genes related to breast/ovarian cancer, we paid particular attention to those harboring the same variant shared by at least three unrelated patients. For instance, the variant c.2645T>C; p.Ile882Thr in the *KMT2C* gene was shared by four unrelated patients; three were ER^+^. This variant was not reported in ClinVar and classified as VUS in Varsome.

The H3K4 methyltransferase *KMT2C* is a critical regulator of hormone-dependent ERα activity [52]. Additionally, KMT2C is one of the most frequently mutated genes in ER-positive breast cancer and its loss disrupts proliferation through estrogen and conversely promotes tumor outgrowth in hormone-depleted conditions [52]. Based on the TCGA data (https://www.cbioportal.org/ (accessed on 26 August 2021)), we showed that *KMT2C* was significantly associated with overall survival in patients with BC. Similarly, Gala et al. reported that patients with *KMT2C* mutations had a lower progression-free survival rate on hormone deprivation therapy than patients with wild-type *KMT2C*, suggesting the probable contribution of KMT2C to BC hormone resistance [52].

RHPN2, a RhoA-binding protein, promotes malignant cell proliferation in ovarian cancer by activating the JAK2/STAT3 signaling pathway [53]. Here, we identified in four unrelated patients the same splicing variant (c.1225+5G>A) that could impact the splicing as predicted by in silico tools.

FOXP2, a member of the forkhead box transcription factor family, is suggested to regulate the progression of cancer cells through the epithelial–mesenchymal transition [54]. Keeping in mind that the variation in the 3′UTR region plays an important role in gene regulation [55,56], we identified in three unrelated patients, a variant in the 3′UTR region that could affect the expression of FOXP2, but further investigations are needed to better understand the effect of these variants.

HSPG2, also known as perlecan, is a heavily glycosylated protein component of the extra-cellular matrix (ECM) that has been previously observed as part of the surface of malignant cells of various cancers [57]. A strong correlation between HSPG2 expression and survival of TNBC patients was reported, suggesting that HSPG2 might be a promising therapeutic target in metastatic TNBC [58].

Cytokines are a protein family of regulatory factors derived from tumors and their environmental components that contribute to the growth, invasion, and metastasis of cancer. CCR3, a receptor of the C-C motif chemokine ligand (CCL), is identified to be one of the major factors that is involved in the progression and metastasis of some human tumors [59,60,61,62]. Recently, Yamaguchi et al. demonstrated that the CCL5–CCR3 interaction contributed to tumor progression suggesting that this axis may be an important target to improve the prognosis of breast cancer patients [63]. 

Finally, we associated the 24 selected genes with patients survival using TCGA data. Kaplan–Meier curves showed that *EP300*, *KMT2C*, *RHPN2*, *HSPG2*, and *CCR3* genes correlated significantly with overall or disease-free survival rates.

## 5. Conclusions

In the present study, we selected 24 candidate genes form Tunisian patients with HBOC through functional and bioinformatic analyses of exome sequencing data. Regarding their role in carcinogenesis and their contribution to key signaling pathways, we retained a short list of five potential new candidate genes associated with HBOC in non-*BRCA* mutation carriers, namely, *EP300*, *KMT2C*, *RHPN2*, *HSPG2*, and *CCR3.* Nevertheless, further studies based on co-segregation analyses of affected families and the locus-specific loss of heterozygosity are needed to highlight the relevance of these candidate genes for HBOC risk.

## Figures and Tables

**Table 1:** Clinico-pathological parameters of patients selected for exome sequencing.

Patients	Age at Diagnosis	Family History of BC/OC	Family History of Other Cancers	Histological Type	SBR Grade	Tumor Size	TNM	ER	PR	Her-2	Therapy	Follow-Up
BC-F2P10	30	9BC, 2MBC, 3OC	-	IDC	II	2	I	-	-	-	TAM, FEC, TXT, RT	NA
BC-F7P1	37	3BC, 1OC	Prostate	IDC	II	3	IIB	+	+	-	MCA, TAM, FEC, TXT, RT	Metastasis after 3 y of BC. Died with the disease progression
BC-F10P6	27	7BC	Endometrial	IDC	III	4.5	IIA	+	+	-	MCA, TAM, FEC, TXT, RT	Remission with OS of 1 y
BC-F11P4	30	4BC, 1OC	Prostate	IDC	III	5.3	IIB	-	-	-	FEC, RT	Remission with OS of 16 y
BC-F12P3	49	4BC, 1OC	Prostate Melanoma	IDC	II	4.5	IIB	+	-	-	MCA, TAM, RT	Remission with OS of 6.5 y
BC-F16P5	24	5BC, 1BC/Thy	Thyroid	IDC	II	2.5	IIA	+	+	-	TAM, FEC, TXT, RT	Remission with OS of 4.5 y
BC-F42P5	30	3BC, 2BC/OC	Prostate	IDC	II	4	IIB	+	+	-	MCA, TAM, FEC, C75, RT	NA
BC-F46P5	55	4BC, 1MBC	-	IDC	II	2	IA	+	+	-	TAM, FEC, TXT, RT	Remission with OS of 1 y

**Table 2:** List of genes commonly associated with hereditary breast/ovarian cancer identified by exome sequencing in each patient.

Patients	Gene	rs	Variation	MAF	Consequence	ClinVar
**BC-F2P10**	*PMS1*	778185859	c.278G>A; p.Arg93His	0.000062	VUS	NR
	*RAD54B*	35973866	c.247A>G; p.(Ser83Gly)	0.0408	VUS	NR
	*ATM*	587781352	c.2492A>G; p.(Asp831Gly)	0.000016	VUS	R
	*RAD51D*		c.413A>G; p.(Asn138Ser)	0.000756	VUS	R
	*EP300*	1268191227	c.3976G>A; p.(Val1326Ile)		P	R
	*MUTYH*	140118273	c.1544C>T; p.(Ser515Phe)	0.0234	B	R
**BC-F7P1**	*ATM*	2227922	c.1810C>T; p.(Pro604Ser)	0.0317	LB	R
	*RAD52*	867412462	c.1048G>A; p.(Asp350Asn))	0.000018	VUS	NR
	*RAD54B*	116312454	c.2639A>G; p.(Asp880Gly)	0.0043	LB	R
**BC-F10P6**	*FANCE*	768911543	c.298T>A; p.(Ser100Thr)	0.00072	VUS	R
	*PMS2*		c.706-4dup		VUS	NR
	*EXO1*	4150001	c.2276G>A; p.Gly759Glu	0.0338	LB	R
	*RAD52*	4987206	c.661C>G; p.Gln221Glu	0.0325	B	R
**BC-F11P4**	*RAD51D*	NR	c.*1056C>T	NA	LB	NR
**BC-F12P3**	*FANCL*	770368316	c.288G>T; p.Lys96Asn	0.000416	VUS	R
	*POLL*	61757734	c.169C>T; p.Arg57Trp	0.0026	VUS	NR
	*POLH*	35675573	c.986C>T; p.Thr329Ile	0.017	LB	R
**BC-F16P5**	*ATM*	864622251	c.6115G>A; p.Glu2039Lys	0.000033	VUS	R
	*DCC*	138724679	c.527A>G; p.Asn176Ser	0.0005	VUS	R
	*BARD1*	61754118	c.2212A>G; p.Ile738Val	0.027	LB	R
	*XRCC1*	143917286	c.818C>T; p.Pro273Leu	0.00311	LB	R
	*RAD52*	4987206	c.661C>G; p.Gln221Glu	0.0325	B	R
	*RAD51D*	NR	c.*106G>A	NA	LB	NR
**BC-F42P5**	*BLM*	141503266	c.254G>C; p.Arg85Thr	0.01	LB	R
	*PMS2*	63750055	c.1711C>A; p.Leu571Ile	0.02	LB	NR
**BC-F46P5**	*EP300*		c.227C>G; p.Ser76Cys		VUS	
	*RAD54B*	2919661	cc.289G>C; p.Asp97His	0.018	VUS	NR

VUS: variant of unknown significance, LB: likely benign, B: benign, R: reported, NR: not reported, NA: not available, MAF: minor allele frequency, *: stop codon.

**Table 3:** List of genes with heterozygous rare stop-gain and frameshift variants identified in each patient. Bold characters indicate common variants in unrelated patients.

Patient	Gene	rs	Variation	MAF	Consequence	ClinVar
**BC-F2P10**	*PCK2*	753706965	c.577c>T; p.Arg193*	0.000415	P	R
	*PDE6B*	NR	c.125_126insTGCGA; p.Asp43Alafs*109	NA	LP	NR
	*PDE6B*	NR	c.120_121insGAGGA; p.Pro41Glufs*111	NA	LP	NR
	*IL31RA*	144337484	c.700C>T; p.Arg234*	0.000163	LP	NR
	*TTC37*	768215813	c.1708C>T; p.Arg570*	0.000054	LP	NR
	*SH3PXD2B*	551498843	c.2626_2629del; p.Phe876Lysfs*16	0.005594	P	R
**BC-F7P1**	*ZnHIT6*	NR	c.1114G>T; p.Glu372*	NA	LP	NR
	*BHMT*	763726268	c.1200del; p.Lys400Asnfs*15	0.000746	LP	R
	*MEGF10*	NR	c.122C>A; p.Ser41*	NA	LP	NR
**BC-F10P6**	*BHMT*	763726268	c.1200del; p.Lys400AsnfsTer15	0.000746		R
	*Cyp3A5*	28383469	c.92dup; p.Leu32Thrfs*3	0.011928	LP	NR
	*ALOX15*	781725832	c.316del; p.Leu106*	0.000229	LP	NR
	*SMUG1*	2233919	c.7C>T; p.Gln3*	0.054463	LB	NR
**BC-F11P4**	*MAN1B1*	NR	c.383T>G; p.Leu128*	NA	LP	NR
**BC-F12P3**	*PIKFYVE*	NR	c.914C>A; p.Ser305*	NA	LP	NR
	*IL3*	373251020	c.337C>T; p.Arg113*	0.000056	LP	NR
	*TSSK4*	200353859	c.895A>T; p.Lys299*	0.002882	LP	NR
	*APOBEC3B*	199817842	c.166C>T; p.Arg56*	0.002176	LP	NR
**BC-F16P5**	*CERKL*	121909398	c.847C>T; p.Arg283*	0.000538	P	R
	*PIKFYVE*	NR	c.573C>A; p.Cys191*	NA	LP	NR
	*TM4SF19*	NR	c.273T>A; p.Cys91*	NA	LP	NR
	*ALG1*	NR	c.297_298del; p.Val100Phefs*37	NA	LP	NR
**BC-F42P5**	*SH3PXD2P*	551498843	c.2626_2629del; p.Phe876Lysfs*16	0.00559		R
	*HMSD*	559021231	c.105_120del; p.Asp35Glufs*49	0.013616	LP	NR
**BC-F46P5**	*SPG11*	NR	c.3235G>T; p.Gly1079*	NA	LP	NR

LP: likely pathogen, P: pathogen, LB: likely benign, R: reported, NR: not reported, NA: not available, MAF: minor allele frequency. *: Stop codon, fs: frameshift.

## Data Availability

Data will be available upon request.

## Supplementary Materials

The following supporting information can be downloaded at: https://www.mdpi.com/article/10.3390/genes13081296/s1, Table S1: List of genes included in each of the 5 clusters identified by MCODE analysis, Table S2: List of 24 gene variants selected by integrative analyses (MCODE, GO, and KEGG) and identified in at least 3 unrelated patients from the selected families.

## Abbreviations

**ACMG**	American College of Medical Genetics and Genomics
**B**	Benign
**BC**	Breast Cancer
**BWA**	Burrows- Wheeler Aligner
**DFS**	Disease Free Survival
**ER**	Eostrogen Receptor
**ES**	Exome Sequencing
**GO**	Gene Ontology
**HBOC**	Hereditary Breast Ovarian/Cancer
**KEGG**	Kyoto Encyclopedia of Genes and Genomes
**LB**	Likely Benign
**LOF**	Loss of Function
**LP**	Likely Pathogen
**MAF**	Minor Allele Frequency
**MCODE**	Molecular Complex Detection
**NA**	No Available
**NGS**	Next Generation Sequencing
**NR**	No Reported
**OC**	Ovarian Cancer
**OS**	Overall Survival
**P**	Pathogen
**PF**	Pass Filter
**PPI**	Protein-Protein Interactions
**PR**	Progesteron Receptor
**PVs**	Pathogenic Variants
**SBR grade**	SBR Scarff-Bloom-Richardson (grade)
**TCGA**	The Cancer Genome Atlas
**TNBC**	Triple Negative Breast Cancer
**VUS**	Variant Unknown Significance
**WES**	Whole Exome Sequencing
