# Supplementary material for: The Identification by Exome Sequencing of Candidate Genes in BRCA-Negative Tunisian Patients at a High Risk of Hereditary Breast/Ovarian Cancer
Source: Genes (Basel). 2022 Jul 22;13(8):1296. doi: 10.3390/genes13081296 (PMC9331434; doi:10.3390/genes13081296)
Supplement: Supplementary file 1 [file genes-13-01296-s001.zip › Table S1 supplementary data.pdf]

Table S1: List of genes in each cluster identified by MCODE analysis

Clusters	Genes
**Cluster 1**	*UTRN, FBN1, NID1,COL12A, MMP2, COL2A, COL7A1,COL6A2,LRP2,LAMA1, LAMC2, COL11A2,VCAN, IHH, FGFR3, COL6A3,LAMA5, LRP8, DCN, HSPG2, FLT1.*
**Cluster2**	*PCDH15, FLNA, MYLK, LDB3, OBSCN, TNNI3, MYH11, TTN, MYBPC3, TPM2, MYH14, MYH3.*
**Cluster3**	*SYNE1, MAPT, RFC2, EHMT1, MED13, PER3, CHD6, EP300, RMI1, SMARCA4, SLX4, ATM, GLI1, FUS, KMT5A, XRCC1, AP32, MMP2, HERC2, TXNRD2, RAD51D, KMT2C, KMT2D,ITPR1, STAT1, ATG7, DICER1, NOTCH3, BRD1, IL17A, POLL, FANCL.*
**Cluster4**	*SLC22A1, CYP2C19, ATP8B1, ABCG2, SLC15A1, ABCC2, CYP1A1, ABCB11, HMGCR, CFTR,MYO5B, GLI1.*
**Cluster5**	*DRD5, GRK3, MAOB, CCR3, PDE6B, CHRNA4, PDE11A, GUCY2C, NPR1, NCS1, CNGB1, PRKACA.*
